# Morphological Awareness in L2 Chinese Reading Comprehension: Testing of Mediating Routes

**DOI:** 10.3389/fpsyg.2021.736933

**Published:** 2021-10-13

**Authors:** Haomin Zhang, Xing Zhang, Mengjie Li, Yiming Zhang

**Affiliations:** ^1^The Psycholinguistics Lab, School of Foreign Languages, East China Normal University, Shanghai, China; ^2^School of English Studies, Shanghai International Studies University, Shanghai, China

**Keywords:** logographic writing system, lexical inferencing, morphological awareness, comprehension, mediation, direct and indirect effect, covariate factor

## Abstract

This study aims to examine the contribution of morphological awareness to second language (L2) Chinese reading comprehension through potential mediating factors. Adult L2 Chinese learners (*n* = 447) participated in the study and completed two morphological awareness tasks (segmentation and discrimination), two vocabulary knowledge tasks (character knowledge and word-meaning knowledge), one lexical inference task, and one reading comprehension task. By testing alternative path models, this study identified the preferred model assuming the covariates of morphological awareness and vocabulary knowledge. Morphological awareness and vocabulary knowledge jointly contributed to L2 Chinese reading comprehension through lexical inference. The written modality of morphological awareness induced the activation of both morphological and orthographic information in print. The result suggests that morphological awareness (in the form of grapho-morphological knowledge) and vocabulary knowledge seem to be two parallel components under the same construct predicting Chinese reading comprehension. More importantly, this study underscores the intermediary effect of lexical inference in associating morphological awareness and reading comprehension in L2 Chinese learners.

## Introduction

### Chinese Morphological Awareness

Morphological awareness was broadly defined as the sensitivity to morphemic structures of words and the ability to reflect upon morphological structures (Carlisle, 1995). In the case of taking morphological processes into account, morphological awareness incorporates different sets of cognitive and linguistic capabilities, such as segmentation, recognition, discrimination, and production (Tyler and Nagy, 1989; Koda, 2000; Mcbride-Chang et al., 2005).

Chinese morphology is characterized by lexical compounding given that modern Chinese does not mark tenses or parts of speech morphologically (Sun, 2006), and compounding is the most salient word-formation rule in Chinese (Ceccagno and Basciano, 2007). Corpus-based analyses have indicated that 75–80% of Chinese words are formed by two or more morphemes/characters (Packard, 2000). According to the headedness of lexical compounding (Ceccagno and Basciano, 2007), Chinese compound words can be divided into three major categories: subordinate compounds (e.g., 毒贩, drug + seller, drug trafficker), attributive compounds (天价, sky + price, prohibitive price), and coordinate compounds (e.g., 大小, big + small, size). The processing of Chinese multi-morphemic words starts from the structural segmentation of fundamental orthographic units of words (Taft and Zhu, 1995). The structural and functional information of morphologically complex ones is encoded through individual characters (morphemes). For instance, the meaning of 播音员 (broadcaster) can be activated through structural segmentation and functional mapping. Given the structural regularity and semantic transparency, the word can be broken down into 播音 (broadcast/announce) and 员 (person/professional). The meaning can be retrieved based on each functional element: 播 (broadcast), 音 (sound), and 员 (professional).

In brief, morphological decomposition and discrimination of compound words are the critical competencies involved in Chinese morphological processing. Building structural and semantic connections in morphologically complex compound words tends to be the essence of Chinese morphological awareness.

### Morphological Awareness in Chinese Reading Comprehension: Direct and Indirect Effects

The utility of morphological awareness has been endorsed in reading across languages. Several prior studies explored the facilitative role of morphological awareness in Chinese reading comprehension among the first language (L1) or bilingual children (Wang et al., 2006; Pan et al., 2016; Zhang, 2016a,b; Lin et al., 2018; Zhao et al., 2019; Qiao et al., 2021).

Chinese morphological awareness highlights the language specificity of lexical compounding. Based on the covariance-based evidence, Wang et al. (2006) explored the cross-linguistic contribution of morphological awareness to Chinese–English biliteracy acquisition. Parallel tasks (derivational awareness and compound awareness) were administered to bilingual children. In addition, homophone identification was assessed as an indicator of Chinese-specific morphological awareness. The results demonstrated that cross-linguistic compound awareness made significant contributions to Chinese reading comprehension. Moreover, Pan et al. (2016) emphasized the longitudinal pattern of metalinguistic awareness in Chinese literacy development. Through a cross-lagged analysis, the results confirmed a significant longitudinal impact of morphological awareness on character-level processing (character reading and writing) and higher-level literacy skills (reading comprehension).

Recent studies have emphasized mediated and unmediated effects of morphological awareness on Chinese reading comprehension to unpack the mechanism in which morphological awareness is associated with Chinese higher-level reading ability. There are two strands of findings: full mediation and partial mediation. Pertaining to full mediation, Zhang (2016a) examined the role of morphological awareness in literacy acquisition among Chinese early elementary-age students. Morphological awareness measurements, vocabulary knowledge, lexical inference, and reading comprehension were administered to the students. The results showed that morphological awareness did not contribute directly to Chinese reading comprehension. The contribution was fully mediated by vocabulary knowledge and lexical inference. Zhang (2016b) further explored both concurrent and longitudinal effects of morphological awareness on Chinese reading comprehension among elementary-age students. These findings verified an indirect longitudinal contribution of morphological awareness to reading comprehension through lexical inference.

With regard to partial mediation, Zhao et al. (2019) tested multiple mediation routes of how morphological awareness relates to reading comprehension among Chinese children. Their findings demonstrated that morphological awareness contributed to reading comprehension through character recognition and reading fluency longitudinally. Nonetheless, the mediation through vocabulary knowledge and reading fluency was not significant. Kim et al. (2020) explored multiple alternative path routes by which Chinese compound awareness correlates with reading comprehension among Chinese second graders. The pathways included a direct route and an indirect route *via* word reading, listening comprehension, and vocabulary. The findings revealed that Chinese compound awareness was related to reading comprehension directly and indirectly through the mediation of all tested mediating variables. Similarly, Qiao et al. (2021) analyzed the manner in which morphological awareness contributes to Chinese and English reading comprehension among Chinese children in Grades 3 and 4. The results showed both direct and indirect effects of morphological awareness on Chinese and English comprehension. The indirect effects were mediated through the two path routes: word reading ability (model 1) and vocabulary and word reading (model 2) after control variables were included, which highlighted the significant pathways through word reading. However, the meditation solely through vocabulary was not significant.

A few important points can be summarized from the existing reading studies. First, morphological awareness has been found to predict Chinese reading comprehension directly and indirectly *via* the mediating factors. Second, the mediating routes included the pathways through word reading, reading fluency, listening comprehension, vocabulary knowledge, and lexical inference. Yet, there is a paucity of research examining the role of morphological awareness in second language (L2) Chinese reading comprehension. The current study highlights both direct and indirect contributions of morphological awareness to L2 Chinese reading comprehension. To be more precise, the overarching question focuses on the manner in which morphological awareness is associated with L2 Chinese reading comprehension through potential mediating factors.

## Methods

### Participants

447 English-speaking L2 Chinese learners (312 women and 135 men) from college-level study-abroad programs participated in this study. The age of learners ranged from 18 to 32 (mean age = 22.13, SD = 3.37). They were all college students with a mixture of degree-seeking students and non-degree-seeking language students. At the time of data collection, they had at least one academic year of formal Chinese learning. With the placement tests at their institutions, they all were placed into the courses of intermediate level from low- to high-intermediate level. The researchers obtained the consent of the study-abroad program coordinators before recruiting participants in the classrooms, and the learners volunteered to participate in the study. Participating students were expected to have acquired the basic linguistic competencies of print Chinese as this research probed into various facets of print knowledge. Data were collected in a class session with approximately 15 students in each session. All tasks were randomized in different sessions to eliminate learning effects from prior tasks.

### Measurements

#### Morpheme Segmentation

Morpheme segmentation assessed the ability of learners to break down three-character words into two meaningful lexical morphemes. The participants were expected to segment multimorphemic three-character words into two parts. For example, a multimorphemic word “飞机场 (fly machine-field)” (airport) was shown to the participants. They were asked to draw a vertical line between the characters to break down the bimorphemic word into two components. In this example, the participants ought to draw a line between the morpheme “飞机 (airplane)” and the morpheme “场 (field).” There were 20 items in the segmentation task, including four derivational words and 16 compound words.

#### Morpheme Discrimination

The morpheme discrimination task, modeled after Ku and Anderson (2003), was used to measure the ability of learners to extract partial word information and to distinguish the functional components of morphologically complex words. In this task, three seemingly compound words were shown to the participants, for instance, 小猫 “little cat”, 小狗 “little dog,” and 小心 “careful.” Apparently, these words share the same morpheme “小,” but the word “小心 (careful)” does not bear the meaning of “little.” The participants should select the word whose morphemic meaning was different from the other two meanings. There were 20 items in the morpheme discrimination task.

#### Character Knowledge

The character knowledge test was to assess the ability of learners to extract graphic representations (Chinese characters) of visually presented stimuli (Zhang et al., 2019). The participants were required to choose the most appropriate Chinese character combinations in each stimulus. For example, an English stimulus “the day after tomorrow” was shown with the following four selections: A. 前天 (the day before yesterday), B. 明天 (tomorrow), C. 昨天 (yesterday), and D. 后天 (the day after tomorrow). There were 30 items in the character knowledge task.

#### Word-Meaning Knowledge

This task tapped into the ability of learners to match semantic meanings with visually presented words (Zhang et al., 2019). The participants were required to choose the correct meaning for each word. For instance, the word “导演” was presented to the participants, and they were supposed to select the appropriate explanation from the following items: A. actor, B. director, C. film, and D. designer. These items were chosen from the Hanyu Shuiping Kaoshi (HSK, standardized Chinese test) word lists with ascending difficulty. There were 30 lexical items in the word-meaning knowledge task.

#### Lexical Inferencing Ability

Lexical inferencing ability was designed to assess the performance of learners on semantic retrieval by utilizing word-internal and word-external information. All target words were disyllabic compound words, and each word included two elementary-level characters from HSK levels 1 and 2 (the lowest bands in HSK). However, they were unfamiliar to the participants as all the words were beyond the highest level of HSK. In an initial pilot study, 14 level-appropriate Chinese learners in the USA were asked to assess the familiarity of 20 initially selected words, and 16 compound words were finalized. The lexical inferencing measurement has also been validated in the prior studies of researchers (Zhang and Koda, 2018a; Zhang et al., 2019). In this task, each compound word was placed into a sentence, and the participants were required to derive word meanings by the given information (partial word/morphological information and contextual clues). For example, a sentence “这个球员很有名” (This ___ is very famous) with four options was presented to the participants: A. employee (morphology–, context+); B. player (morphology+, context+, correct); C. ball boy (morphology+, context–); and D. speaking assistant (morphology–, context–). The second option should be chosen if the participants accurately employ the word-internal and word-external clues in the sentence. There were 16 items in this task.

#### Reading Comprehension

The reading comprehension task adopted from the HSK test was to measure the ability of learners to identify coreference, specific contextual information, and main ideas of short passages. Based on the instructional level of participants, reading comprehension questions were selected from HSK levels 3 to 5. There were 18 short passages and 18 follow-up comprehension questions (one question per passage). All questions were followed with four options, and the participants were asked to choose the most appropriate one. Sample items of all six measurements are given in the Supplementary File.

## Results

### Descriptive Statistics and Correlations

Table 1 presents the descriptive results of morphological awareness, vocabulary knowledge, lexical inference, and reading comprehension. The accuracy rates of the tested variables were in the range from 74.2% (word-meaning knowledge) to 81.3% (morpheme segmentation ability). Based on SDs, most of the measurements had adequate dispersions. Vocabulary knowledge measures, including character knowledge and word-meaning knowledge, were relatively widespread. The indices of skewness and kurtosis (based on the raw scores) showed that all the measurements had normal distributions.

**Table 1:** Descriptive statistics of morphological awareness, vocabulary knowledge, lexical inference, and reading comprehension.

**Variable**	**M**	**SD**	**Min**	**Max**	**Skewness**	**Kurtosis**	**Cronbach α**
Morpheme segmentation (20)	16.25	2.62	6	20	−1.10	1.05	0.718
Morpheme discrimination (20)	15.77	3.36	2	20	−1.13	1.08	0.770
Character knowledge (30)	23.80	4.37	4	30	−0.99	1.44	0.763
Word-meaning knowledge (30)	22.25	5.17	4	30	−0.73	0.09	0.816
Lexical inference (16)	12.13	2.74	3	16	−0.87	0.32	0.747
Reading comprehension (18)	14.03	3.56	4	18	−1.15	0.82	0.763

Table 2 shows the correlations among the variables. All the measurements had significant correlations with each other. Morphological awareness in the form of morpheme segmentation and morpheme discrimination had significant and moderate relationships with reading comprehension (*r* = 0.46, *p* < 0.001; *r* = 0.57, *p* < 0.001). Vocabulary knowledge indexed by character knowledge and word-meaning knowledge also had significant and moderate correlations with reading comprehension (*r* = 0.48, *p* < 0.001; *r* = 0.59, *p* < 0.001). Lexical inference had a relatively strong correlation with reading comprehension (*r* = 0.61, *p* < 0.001).

**Table 2:** Bivariate correlations among morphological awareness, vocabulary knowledge, lexical inference, and reading comprehension.

**Variable**	**1**	**2**	**3**	**4**	**5**	**6**
1. Morpheme segmentation	–					
2. Morpheme discrimination	0.51^***^	–				
3. Character knowledge	0.37^***^	0.58^***^	–			
4. Word-meaning knowledge	0.42^***^	0.64^***^	0.78^***^	–		
5. Lexical inference	0.37^***^	0.60^***^	0.53^***^	0.62^***^	–	
6. Reading comprehension	0.46^***^	0.57^***^	0.48^***^	0.59^***^	0.61^***^	–

*** *p < 0.001*.

### Hierarchical Regression

Prior to the model testing, hierarchical multiple regression was employed to confirm the relative contributions of the tested variables to reading comprehension (a scatter plot is provided in the Supplementary File). Morphological awareness, vocabulary knowledge, and lexical inference were subsequently entered into three different blocks. The results demonstrated that morphological awareness predicted a significant proportion of variance in reading comprehension (37.8% of the total variance explained). Both segmentation and discrimination abilities contributed to reading comprehension (β = 0.229, *p* < 0.001; β = 0.467, *p* < 0.001). Vocabulary knowledge explained 6.3% additional significant variance in reading comprehension after morphological awareness was controlled for. To be more precise, word-meaning knowledge had a significant impact on reading comprehension (β = 0.305, *p* < 0.001), whereas character knowledge had non-significant contributions to reading comprehension (β = 0.032, *p* = 0.632). Finally, lexical inference predicted 6.6% extra significant variance in reading comprehension even after the control of morphological awareness and vocabulary knowledge (β = 0.336, *p* < 0.001).

### Identifying Preferred Models

**Figure 1:**
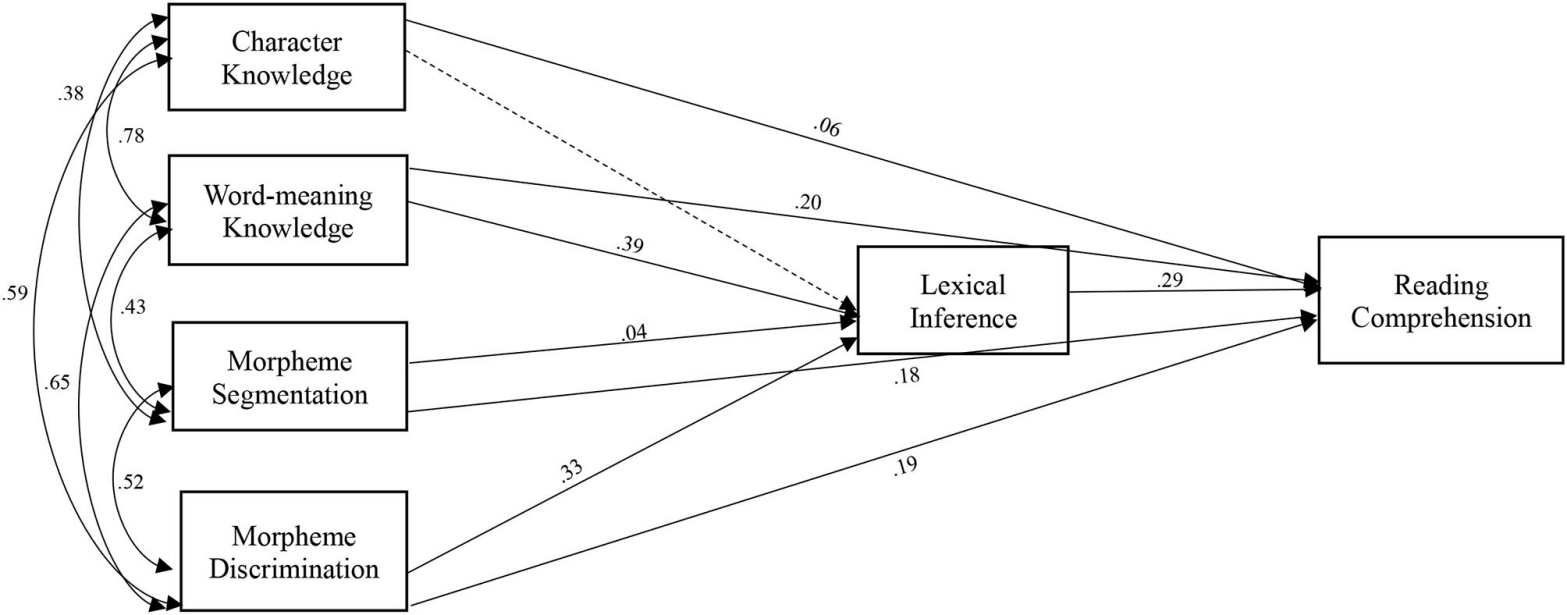
Model 1 hypothesizing the joint contribution of morphological awareness and vocabulary knowledge to reading comprehension through lexical inference. The dotted line was removed in the model re-specification.

**Figure 2:**
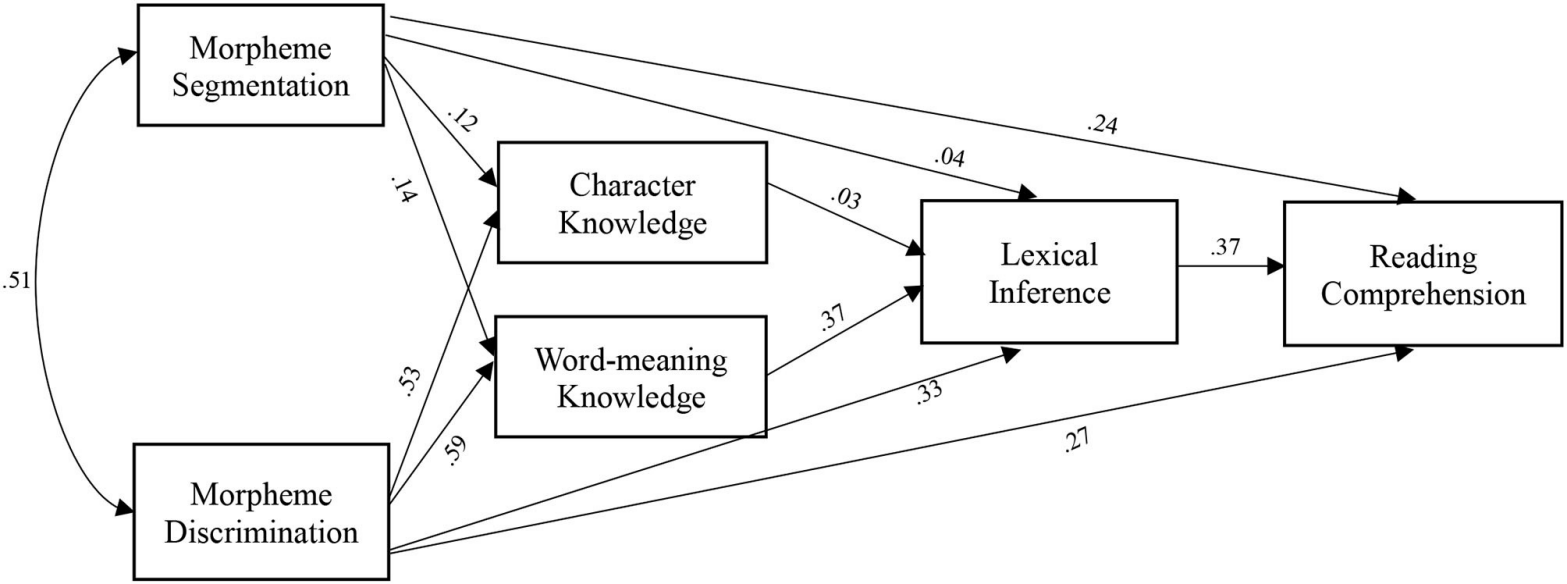
Model 2 hypothesizing the mediation through vocabulary knowledge and lexical inference.

A path analysis was then conducted to explore the interconnected relationship among morphological awareness, vocabulary knowledge, lexical inference, and reading comprehension in L2 Chinese learners. Two alternative path models were tested. The first model hypothesized that morphological awareness and vocabulary measures were the covariates that predicted reading comprehension *via* lexical inference (Figure 1). The second model assumed that morphological awareness predicted reading comprehension through vocabulary knowledge and lexical inference (Figure 2). By testing the goodness-of-fit measures, we specified and identified a preferred path diagram. Model fit indices were initially computed based on the diagram that all the variables are connected. Given the degrees of freedom, re-specification was conducted in both models. In both models, character knowledge did not yield significant contributions to lexical inference; therefore, the route between character knowledge and lexical inference was removed in the model fit analysis.

A few studies on applied statistics and psychometrics suggest that comparative fit index (CFI), normed-fit index (NFI), non-NFI (NNFI) > 0.95 (Bentler, 1990), and a root mean square error of approximation (RMSEA) < 0.08 (Browne and Cudeck, 1993) yield a good model fit. According to the model fit indices, the first model had a perfect model fit $\chi_{\left(1,\text{n}=447\right)}^{2}$ = 0.294, *p* = 0.588 (CFI = 1.00, NFI = 1.00, NNFI = 1.00, RMSEA = 0.00), whereas the second model did not yield an acceptable fit: $\chi_{\left(4,\text{n}=447\right)}^{2}$ = 238.57, *p* < 0.0001 (CFI = 0.811, NFI = 0.811, NNFI = 0.813, RMSEA = 0.158). Therefore, the model assuming the covariates of vocabulary knowledge and morphological awareness in predicting reading comprehension was identified as the preferred model.

### Testing of Direct and Indirect Effects

Model 1 was identified as the path model to further examine the direct and indirect effects of morphological awareness on reading comprehension. Table 3 presents the standardized regression weights of individual variables. In the model fit testing, morphological awareness and vocabulary knowledge measures were found to be the covariates predicting reading comprehension. The model estimates of the path diagram showed that the morphological awareness measures (morpheme segmentation and morpheme discrimination) and word-meaning knowledge had significant direct contributions to reading comprehension ($\hat{\beta }$ = 0.176, *p* < 0.001, $\hat{\beta }$ = 0.193, *p* < 0.01, $\hat{\beta }$ = 0.203, *p* < 0.01), whereas character knowledge did not contribute to reading comprehension ($\hat{\beta }$ = 0.055, *p* = 0.40). Through a mediating route of lexical inference, we found that morpheme discrimination and word-meaning knowledge had significant contributions to lexical inference ($\hat{\beta }$ = 0.334, *p* < 0.001, $\hat{\beta }$ = 0.387, *p* < 0.001). Meanwhile, the connection between lexical inference and reading comprehension was significant ($\hat{\beta }$ = 0.290, *p* < 0.001).

**Table 3:** Standardized regression weights for a preferred path model.

**Paths**			**β**	***S.E*.**	***C.R. (z)***	***p***
Lexical inference	< –	Word-meaning knowledge	0.387	0.025	8.31	0.000
Lexical inference	< –	Morpheme discrimination	0.334	0.040	6.73	0.000
Lexical inference	< –	Morpheme segmentation	0.042	0.044	0.99	0.325
Reading comprehension	< –	Morpheme segmentation	0.176	0.070	3.53	0.000
Reading comprehension	< –	Word-meaning knowledge	0.203	0.053	2.76	0.006
Reading comprehension	< –	Morpheme discrimination	0.193	0.067	3.13	0.002
Reading comprehension	< –	Lexical inference	0.290	0.075	5.23	0.000
Reading comprehension	< –	Character knowledge	0.055	0.056	0.84	0.401

In summary, character knowledge had no significant direct or indirect contributions to reading comprehension. Morphological awareness had a direct contribution to reading comprehension. Finally, it is worth noting that morphological discrimination and word-meaning knowledge contributed to reading comprehension through a partial mediation of lexical inference.

## Discussion

The study yielded two sets of important findings. First, instead of assuming the developmental sequence of morphological awareness and vocabulary, the preferred model verified that the two constructs were the covariates in predicting L2 Chinese reading comprehension. Second, morphological awareness facets had both direct and indirect effects on L2 reading comprehension through the mediation of lexical inference.

### Joint Contributions of Morphological Awareness and Vocabulary Knowledge to L2 Chinese Reading Comprehension

One salient finding of the current study was the joint and non-sequential contribution of morphological awareness and vocabulary knowledge to L2 Chinese reading comprehension. Prior studies tested a few pathways assuming the developmental order of morphological awareness and vocabulary in L1 Chinese reading comprehension (Zhang, 2016a; Zhao et al., 2019; Kim et al., 2020; Qiao et al., 2021). Nevertheless, the current study found that morphological awareness and vocabulary knowledge were the two parallel components in predicting L2 Chinese reading comprehension. There are a few interpretations based on the existing literature. First, the modality of vocabulary knowledge may affect the developmental pattern of morphological awareness and vocabulary knowledge in reading. Most previous studies capitalized on young students' orally based morphological awareness and vocabulary (e.g., Zhao et al., 2019; Kim et al., 2020). Oral language constructs the initial linguistic competencies for advanced reading acquisition (Ouellette, 2006; Foorman et al., 2015). According to the extant literature, orally assessed morphological awareness enhanced oral vocabulary, thus contributing to reading comprehension in L1 Chinese. However, this study measured the written morphological awareness and vocabulary knowledge in L2 Chinese. In addition to the oral morphological analysis, visually presented stimuli induced grapho-morphological activation of target words. The written vocabulary also incorporates partial word-meaning activation. Therefore, the task modality of morphological awareness and vocabulary may not generate the potential causal pattern of the two constructs. Second, the discrepancy between L1 Chinese reading and L2 Chinese reading may attribute to the results. Metalinguistic awareness develops as long as learners are found to have exposure to oral and written communication (Berko, 1958). Morphological awareness initially developed through the constant input in L1 enhances vocabulary acquisition (Mcbride-Chang et al., 2005). Metalinguistic awareness is a byproduct of early vocabulary. Zhang and Koda (2018b) scrutinized the contributions of vocabulary knowledge and morphological awareness to Chinese heritage language reading comprehension. Through structural equation modeling with a bootstrap estimation method, the study demonstrated that both vocabulary knowledge and morphological awareness contributed to L2 Chinese reading comprehension. The bootstrap estimation verified the precedent role of vocabulary knowledge in Chinese reading comprehension. In other words, vocabulary knowledge contributed to morphological awareness, which in turn enhanced Chinese reading comprehension. Like L1 readers that have early exposure to Chinese, heritage language students can exploit extant vocabulary knowledge to enhance metalinguistic awareness, and subsequently reading comprehension. Nonetheless, in the L2 context, vocabulary knowledge and morphological awareness both are emerging capacities given that learners have limited experiences in the target language prior to the formal classroom instruction. Insufficient vocabulary knowledge may not benefit the abstraction of morphological structures and morphemic meanings. Our study lent support to the codevelopment of morphological awareness and vocabulary knowledge in L2 Chinese reading comprehension.

### Direct and Indirect Effects of Morphological Awareness and Vocabulary Knowledge on L2 Chinese Reading Comprehension

As synthesized in the review literature prior studies have confirmed both direct and indirect effects of morphological awareness on reading comprehension among L1 Chinese children. This study emphasized the contribution of morphological awareness and vocabulary knowledge facets to L2 Chinese reading comprehension. First, it is worthy of note that morphological awareness, in the form of morpheme segmentation and morpheme discrimination as well as word-meaning knowledge, had significant direct contributions to Chinese reading comprehension, whereas character knowledge had no direct contributions to reading comprehension. Although meaning activation was involved, the measurement of character knowledge itself was to capture the ability to understand the orthographic forms of Chinese words. Form- and meaning-induced abilities in word processing seem to generate various degrees of contribution to reading comprehension (Zhang et al., 2019). Second, given the covariates of morphological awareness and vocabulary knowledge, this study verified a mediating effect through lexical inference. The inclusion of vocabulary knowledge in the mediating path routes does not yield significant patterns (Zhao et al., 2019; Qiao et al., 2021); however, reading fluency and word reading ability take the leading roles in connecting morphological awareness and reading comprehension in Chinese. The present findings further underscored the importance of lexical inference in associating morphological awareness and reading comprehension in L2 learners. Considering the sequence from the local word-meaning retrieval to text-meaning construction, we conjecture that morphological awareness constructs local semantic meaning within morphologically complex words and that lexical inference builds upon local semantic abstraction and activation to further retrieve contextual meaning. Such intermediary facilitation ultimately enhances reading comprehension (Zhang, 2016a; Levesque et al., 2017, 2019).

## Conclusions and Implications

This study confirmed the facilitative effect of morphological awareness on L2 Chinese reading comprehension. More importantly, grapho-morphological awareness and written vocabulary knowledge were found to be the two parallel components contributing to L2 Chinese reading comprehension *via* the mediation of lexical inference.

There are also a few limitations, which need further exploration. First, the current study did not administer a standardized proficiency test to control for the proficiency level of learners. Future studies should consider the proficiency control and the moderation effect of proficiency on the correlational pattern. Second, additional socio-cognitive factors may also be the covariates in L2 Chinese reading development. Future studies can incorporate learning motivation/attitudes, learning contexts, and the factors of individual differences.

## Data Availability Statement

The original contributions generated for the study are included in the article/Supplementary Material, further inquiries can be directed to the corresponding author/s.

## Ethics Statement

The studies involving human participants were reviewed and approved by the IRB office of Carnegie Mellon University (Protocol number: HS15-296). The patients/participants provided their written informed consent to participate in this study.

## Author Contributions

HZ contributed to conception and design of the study, performed the statistical analysis, wrote the first draft of the manuscript, and wrote sections of the manuscript. XZ, ML, and YZ organized the database. All authors contributed to manuscript revision, read, and approved the submitted version.

## Conflict of Interest

The authors declare that the research was conducted in the absence of any commercial or financial relationships that could be construed as a potential conflict of interest.

## Publisher's Note

All claims expressed in this article are solely those of the authors and do not necessarily represent those of their affiliated organizations, or those of the publisher, the editors and the reviewers. Any product that may be evaluated in this article, or claim that may be made by its manufacturer, is not guaranteed or endorsed by the publisher.
