# Supplementary material for: Morphological Awareness in L2 Chinese Reading Comprehension: Testing of Mediating Routes
Source: Front Psychol. 2021 Oct 13;12:736933. doi: 10.3389/fpsyg.2021.736933 (PMC8548468; doi:10.3389/fpsyg.2021.736933)
Supplement: Supplementary file 1 [file Data_Sheet_1.docx]

**Scatter Plot of Measurements**


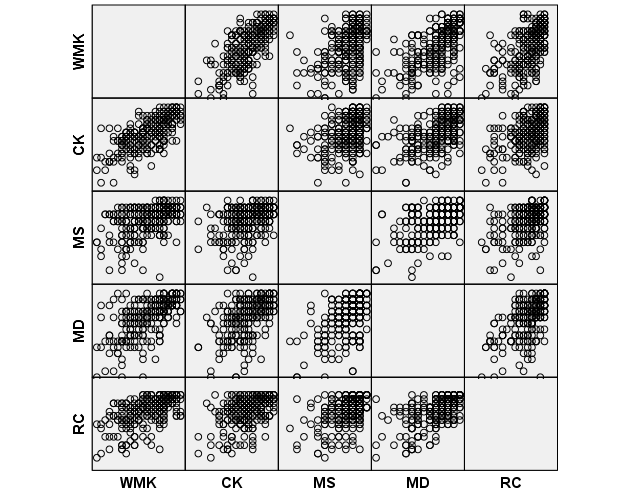


*WMK* Word-meaning knowledge, *CK* Character knowledge, *MS* Morpheme segmentation, *MD* Morpheme discrimination, *RC* Reading comprehension

Note: horizontal and vertical coordinates represent the mean score of each measurement

**Sample Items**

**Chinese Word-meaning Knowledge**

Please choose the most appropriate meaning based on each character combination

| 1. | 可怜 | A. miserable | B. weird | C. but | D. lovely |
| --- | --- | --- | --- | --- | --- |
| 2. | 公寓 | A. apartment | B. dorm | C. living room | D. studio |
| 3. | 职员 | A. assistant | B. account | C. bank | D. employee |
| 4. | 服装 | A. pants | B. skirts | C. apparel | D. shirts |
| 5. | 导演 | A. actor | B. director | C. film | D. designer |
| 6. | 搭乘 | A. take (transports) | B. take out (money) | C. result in (loss) | D. dress up |
| 7. | 精神 | A. tissues | B. spirits | C. positive | D. negative |
| 8. | 根据 | A. despite | B. however | C. according to | D. thanks to |
| 9. | 抱怨 | A. hug | B. complain | C. discuss | D. explain |
| 10. | 守时 | A. late | B. delay | C. punctual | D. recent |

**Chinese Character Knowledge**

Please choose the most appropriate character representation based on each English meaning

| 1. | Problem | A.问题 | B. 问答 | C. 奇怪 | D. 回答 |
| --- | --- | --- | --- | --- | --- |
| 2. | The day after tomorrow | A. 前天 | B. 明天 | C. 昨天 | D. 后天 |
| 3. | Review | A. 预习 | B. 练习 | C. 复习 | D. 课习 |
| 4. | Tell | A. 高兴 | B. 高速 | C. 告诉 | D. 提醒 |
| 5 | Place | A. 地铁 | B. 地方 | C. 地情 | D. 地势 |
| 6. | Complete | A. 完美 | B. 顽强 | C. 完胜 | D. 完成 |
| 7. | Forgive and understand | A. 原谅 | B. 忘记 | C. 谅解 | D. 体会 |
| 8. | Enjoy and appreciate | A. 性情 | B. 欣赏 | C. 案情 | D. 欣喜 |
| 9. | Seek for | A. 讯息 | B. 信息 | C. 寻找 | D. 询问 |
| 10. | Friendship | A. 友情 | B. 朋友 | C. 客人 | D. 好客 |

**Chinese Morpheme Segmentation**

**Directions:** Below are 20 pairs of words. Please break down each word into two meaningful parts. For example, 工程师----工程|师（engine|er）

| 安全带 | 白头发 | 行李员 | 女孩子 |
| --- | --- | --- | --- |
| 计算机 | 小吃店 | 好客人 | 出租车 |
| 取款卡 | 吸引人 | 交叉口 | 信用卡 |
| 音乐师 | 非常人 | 飞机场 | 汽车站 |
| 市中心 | 时间表 | 主卧室 | 羽毛球 |

**Chinese Morpheme Discrimination**

**Directions:** The three words in each group share a part. In two words, the parts have about the same meaning. Circle the word with the part that has a DIFFERENT meaning.

Example: mushroom bedroom classroom; 左脑，右脑，电脑

| 1. 学者 读者 或者 | 2. 年糕 年级 年轻 | 3. 好吃 好看 好客 | 4. 画家 搬家 作家 |
| --- | --- | --- | --- |
| 5. 同学 文学 数学 | 6. 红花 梅花 天花 | 7. 大楼 大厦 大家 | 8. 海鱼 海边 海报 |
| 9. 开门 开心 开关 | 10. 打球 打鼓 打算 | 11. 美金 美国 美丽 | 12. 拿手 左手 举手 |
| 13. 时间 夜间 房间 | 14. 可怜 可是 可怕 | 15. 如果 白果 水果 | 16. 人才 天才 刚才 |
| 17. 千米 小米 白米 | 18. 小猫 小狗 小心 | 19. 老人 老虎 老王 | 20. 足球 篮球 地球 |

Chinese Lexical Inference

**Please guess the meaning of each underlined word**

1. 我很喜欢吃 日料 。

A. Chinese cuisine B. Japanese food C. daily material D. Korean tissues

2. 他是一个钢琴 高手 。

A. teacher B. high-handed C. expert D. left hand

3. 年纪大了，越来越__眼花___了。

A. visually blurred B. slightly deaf C. eyes with flowers D. mental

4. 我觉得这个男人的__度量__很大。

A. muscular strength B. power of breathing C. bearing D. humor

5. 这件事情是__无果___的。

A. successful B. in vain C. no fruit D. seedless grape

1. 去中国的___旅费___好像很贵。

A. air ticket B. trips with trouble C. flight attendants D. travel expenses

7. 考试被老师给____简化____了。

A. simple chemistry B. fail C. make it simple D. design

8. 我们坐____高铁____去北京。

A. maglev B. high-speed train C. tall building D. high iron

Chinese Reading Comprehension

**Please complete multiple-choices comprehension questions after each short passage**

1．太阳从西边出来了吗？他今天怎么这么早就起床了？他一般都要睡到 8 点 以后才起床。

★ 根据这段话，可以知道今天： A 他今天起得早 B 今天天气不错 C 他今天工作很认真

2．手机使我们的学习、工作越来越方便，除了打电话、写短信外，很多手机 还可以照相，有时候真的方便极了。

★ 手机经常被用来： A 写字 B 发短信 C 做练习

3．“再见”是一个很有意思的词语。“再见”表示“再一次见面”，所以人们离 开时说“再见”，其实也是希望以后再见面。

★ 什么时候说“再见”？ A 关灯 B 见面 C 离开

4．到了机场，他发现护照不见了，在行李箱里找了半天，也没找到，很着急。

★ 他为什么着急？ A 迟到了 B 忘记拿机票了 C 找不到护照了

5．人们经常说：“面包会有的，牛奶也会有的。”是的，如果努力，什么都会 有的。

★ 这句话主要想告诉我们： A 要相信别人 B 兴趣最重要 C 努力才有希望

6．上个星期和朋友们去游泳，把我累坏了，到现在我的腿还在疼。看来我是 应该多锻炼锻炼了。

★ 他打算： A 去医院 B 锻炼身体 C 下午去游泳

7．过去人们喜欢看报纸，现在越来越多的人喜欢在电脑上看新闻。除了看新 闻，人们还可以在网上听歌、看电影、买卖东西。

★ 上网后，人们可以： A 做米饭 B 坐地铁 C 买东西

8．超市里一箱牛奶如果卖 32.56 元，也就是 32 块 5 角 6 分，那可能会带来许 多不方便，因为现在人们的钱包里很少有“分”这么小的零钱。

★ 人们的钱包里很少有： A 6 分 B 5 角 C 2 元

9．刷牙的时候，水太冷或者太热，都会给牙的健康带来不好的影响。研究发 现，用 35 度的温水刷牙才是最合适的。

★ 刷牙时，我们应该： A 使用温水 B 常换牙刷 C 早晚各一次 D 至少刷 5 分钟

10．这种葡萄酒，不仅味道好，而且每个酒瓶也都像一件高级艺术品。很多人 愿意出高价购买它，很多时候是被那些特别的酒瓶吸引了。

★ 这种葡萄酒： A 比较甜 B 是艺术品 C 酒瓶很特别 D 是当地制造的
